# A novel nonsense mutation c.747C>G in the *NEUROD1* gene detected within a Chinese family affected by maturity‐onset diabetes of the young type 6

**DOI:** 10.1111/1753-0407.13607

**Published:** 2024-09-12

**Authors:** Yuwen Li, Qian Wen, Huige Shao, Meng Hao, Yihu Sun, Ting Liu

**Affiliations:** ^1^ Department of Endocrinology The Changsha Central Hospital Affiliated to University of South China Changsha China

**Keywords:** diabetes mellitus, gene sequence, maturity‐onset diabetes of the young type 6, mutation, *NEUROD1*

## Abstract

Highlights

- Maturity‐onset diabetes of the young type 6 (MODY6) is a rare form of monogenic diabetes mellitus due to *NEUROD1* gene mutation on chromosome 2q32.
- A 21‐year‐old woman exhibiting weight loss, polyuria, and hyperglycemia was initially misdiagnosed with type 1 diabetes mellitus.
- Considering the early‐onset age, a three‐generation family history of diabetes, and negative autoimmune antibodies, a MODY diagnosis was suspected.
- Genetic analysis revealed that she inherited a novel heterozygous nonsense *NEUROD1* mutation c.747C>G (p.Tyr249*) from her father.
- Correct MODY6 diagnosis facilitates appropriate interventions.

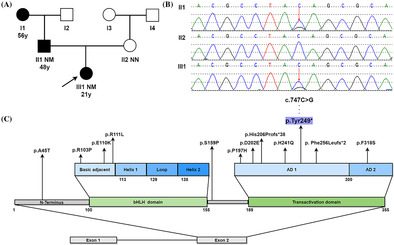

## 1 INTRODUCTION

Maturity‐onset diabetes of the young (MODY) is a monogenic form of diabetes characterized by autosomal dominant inheritance, resulting in pancreatic beta‐cell dysfunction. It constitutes approximately 1%–5% of all diabetes cases. Depending on genetic techniques, there are 14 MODY subtypes based on the different causative gene mutations that have been identified so far. MODY1, MODY2, and MODY3 collectively account for over 90% of MODY cases.^1^ In contrast, MODY6 is exceedingly rare with less than 15 cases reported in the literature.

MODY6 is caused by inactivating mutations in the neurogenic differentiation factor 1 (*NEUROD1*) gene, which is associated with normal pancreatic development and insulin (INS) gene expression. The human *NEUROD1* gene, belonging to the basic helix–loop–helix (bHLH) family, is located at 2q32 and encodes a type II bHLH transcription factor primarily expressed in pancreatic islet endocrine cells, the intestine, and neurons.^2^ The *NEUROD1* protein has the capacity to heterodimerize with the ubiquitous type I bHLH protein E47. It regulates INS gene expression by binding to the E‐box motif promoter in pancreatic beta cells and thereby maintains normal glucose homeostasis.^3^ Until now, a total of 206 single‐nucleotide polymorphisms (SNPs) have been documented in the *NEUROD1* gene, with rs149703259 and rs104893649 being unequivocally identified as pathogenic variants associated with MODY6. Most SNPs identified in reported MODY6 cases are missense or frameshift mutations, with nonsense mutations being exceptionally rare.

In this report, we have revealed a novel heterozygous nonsense mutation in the *NEUROD1* gene within a Chinese family affected by MODY6. Upon genetic testing, the proband was diagnosed with MODY6, received a suitable treatment, and achieved well glycemic control.

## 2 CASE REPORT

A 21‐year‐old Chinese female was admitted to our hospital due to weight loss and polyuria for 1 year and hyperglycemia for 1 month. She was diagnosed with type 1 diabetes mellitus (T1DM) and initiated insulin therapy, but experienced poorly controlled glycemic levels. Physical examination indicated a blood pressure of 115/64 mmHg, a body mass index of 19.5 kg/m^2^, and a waist‐hip ratio of 0.722. Biochemical analyses are as follows: fasting blood glucose: 13.62 mmol/L; 2‐h postprandial blood glucose: 22.51 mmol/L; fasting C‐peptide: 0.3 nmol/L; 2‐h postprandial C‐peptide: 0.68 nmol/; Glycosylated hemoglobin (HbA1c): 11.6%, and urinary microalbumin creatinine ratio 24.2 mg/g. The positive serum ketone and normal arterial blood gas analysis indicated diabetic ketosis. Diabetic autoimmune antibodies were negative. Lipids, liver, and renal function were normal. No abnormalities were detected in electroneuromyography or fundus photography. After hospitalization, she received intravenous fluid infusion and insulin pump, and then, hydroelectrolytic and metabolic disturbances were corrected.

**FIGURE 1:**
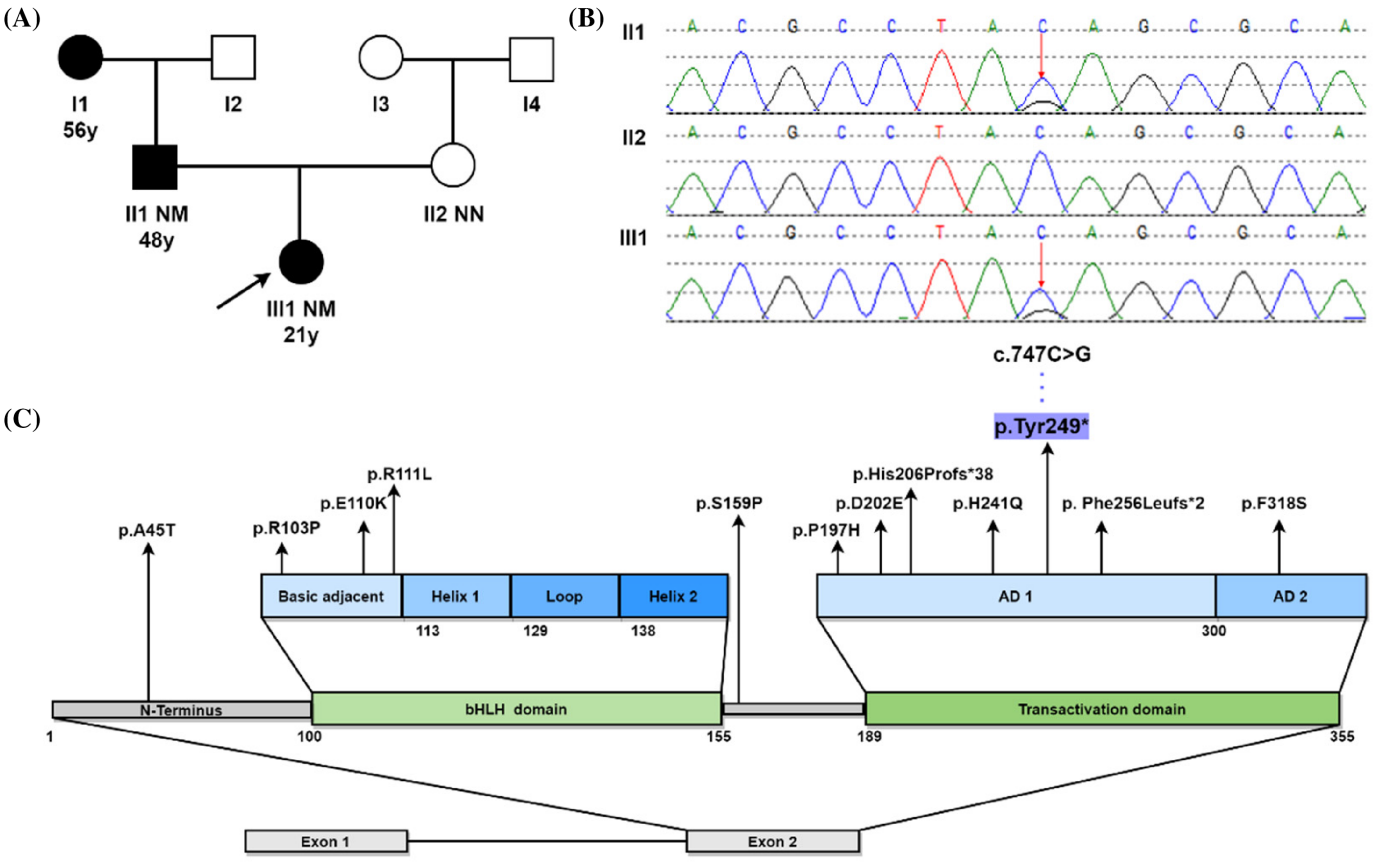
(A) Proband's family tree: Squares represent males and circles represent females. The black symbol represents individuals with diabetes, the blank symbol represents normal individuals. The arrow indicates the proband (III1). Variant carrier status is presented as NN: normal allele and NM: heterozygous *NEUROD1* mutations c.747C>G. The second line under the symbols corresponds to the onset age of diabetes. (B) Gene sequencing results of the proband and her parents. III1 (proband) and II1 (father) both presented heterozygous. (C) *NEUROD1* mutations previously identified in diabetic patients and the novel c.747C>G (p.Tyr249*) mutation. *NEUROD1* gene presents two exons, exon 1 being noncoding. *NEUROD1* protein contains two domains: basic helix–loop–helix (bHLH) domain, which is divided in basic adjacent, helix 1, loop, and helix 2; and transactivation domain, which has two activating domains (AD1 and AD2). Arrows indicate the position of mutations described in the literature and in this study.

The proband's father and paternal grandmother had already been diagnosed with diabetes mellitus at 56 and 48 years old, respectively. Oral Glucose Tolerance Test (OGTT) showed that none of the other relatives in the proband's family had glucose tolerance impairment or diabetes (Figure 1A).

Proband exhibited early‐onset age, partial pancreatic function, negative autoimmune antibodies and a three‐generation family's history of diabetes, a MDOY diagnosis was considered. The peripheral blood of proband and her parents was collected to undergo molecular genetic investigation of 14 MODY‐related genes. Sanger sequencing was used to confirm pathogenic variant locations identified by next‐generation sequencing. Genetic testing revealed that a novel heterozygous *NEUROD1* gene mutation c.747C>G (p.Tyr249*) presented in proband and her father, but not in her mother (Figure 1B). This variant was not seen in dbSNP, HGMD®, ClinVar, 1000 Genomes project databases, or in the existing literature. Based on the interpretation guidelines developed by the American College of Medical Genetics and Genomics to classify the pathogenicity of the variant, the novel *NEUROD1* mutation identified in this family was considered to be “pathogenic,” based on the evidence framework of PVS1 (pathogenic very strong; i.e., a null variant [nonsense]), PM2 (pathogenic moderate; i.e., not found in polymorphism databases), and PP3 (pathogenic supporting; i.e., multiple lines of computational evidence support a deleterious effect on the gene or gene product [MutationTaster score: 1; VEST4 score: 0.933; Likelihood Ratio Test score: 0; and BayesDel score: 0.625]). This indicated the Chinese family suffered from MODY6 due to a variation (c.747C>G, p.Tyr249*) in the *NEUROD1* gene.

Based on the genotyping results, her treatment was adjusted to glimepiride sustained‐release tablet 4 mg once daily. The HbA1c level ranged from 5.9% to 6.7% during follow‐up. Currently, satisfactory glycemic control was maintained, without severe diabetic complications.

## 3 DISCUSSION

Through literature review, only a few *NEUROD1* variants have been documented including A45T, R103P, E110K, R111L, S159P, P197H, D202E, His206Profs*38, H241Q, Phe256Leufs*2, and F318S (Figure 1C). The majority of *NEUROD1* variants occur in the transactivation domain highly conserved across several species.^4^, ^5^, ^6^ Here, we identified a novel nonsense *NEUROD1* mutation c.747C>G (p.Tyr249*) in transactivation domain within a Chinese family. Genetic analysis revealed that a cytosine (C) replaced by a guanine (G) resulting in a change from tyrosine to stop codon at position 249 of the protein, followed by premature translation termination, lack of subsequent amino acid sequence, and truncation of the encoded protein. *NEUROD1* protein consists of two domains, the bHLH domain, which is responsible for DNA binding, and the transactivation domain, which comprises two distinct activation domains (ADs): AD1 and AD2, and contributes to activate multiple gene transcription.^7^ Studies have shown that *NEUROD1* lacking AD2 retains 37% of its activity, but when both AD1 and AD2 are absent, only 10% of the activity is preserved.^5^ This mutation occurred in the AD1 region, resulting in mostly partial deletion of transactivation domain and reducing the binding activity of *NEUROD1* to down‐stream genes including INS gene, glucokinase, and pancreatic and duodenal homeobox factor‐1.^8^ Consequently, proband experienced reduced insulin synthesis and impaired beta cell function, leading to early‐onset hyperglycemia (MODY6). The H241Q, Phe256Leufs*2, and His206Profs*38 variants that are close to this variant also have similar pathogenic molecular mechanisms.^5^

The heterozygous *NEUROD1* mutations demonstrate incomplete penetrance of the phenotype, exhibiting significant heterogeneity among individuals with MODY6 regarding clinical manifestations, age of diagnosis, BMI, and treatments. Some MODY6 patients may not exhibit evident diabetes symptoms, yet the proband presented not only with weight loss and polyuria but also with diabetic ketosis. Notably, episodes of diabetic ketosis have been observed exclusively in carriers with transactivation domain mutations, consistent with the proband in this case. The age of MODY6 diagnosis varies widely, but it is mostly before the age of 25. Consequently, a proportion of young patients are misdiagnosed with T1DM. Over 50% of MODY6 patients with *NEUROD1* mutations present elevated BMI or are obese. The majority of European MODY6 patients are overweight, whereas in China and Japan,^9^ none of the family members (including the proband and her father) exhibit obesity. This suggests that obesity in MODY6 patients may be associated with ethnicity. Based on the review, current clinical treatments for MODY6 include oral hypoglycemic agents and insulin.^10^ *NEUROD1* acts as a pancreatic‐specific transcriptional activator, playing a key role in insulin secretion regulation by modulating the expression of the SUR1 gene. SUR1, a member of the Adenosine triphosphate (ATP)‐binding cassette superfamily, is involved in forming the β‐cell specific ATP‐sensitive K^+^ channel (K_ATP_). In pancreatic β‐cells, increased glucose metabolism inhibits K_ATP_ channel, leading to the activation of voltage‐dependent Ca^2+^ channels and induction of insulin secretion. Study indicates that knockout of the SUR1 gene in mice results in a lack of glucose‐stimulated insulin secretion.^11^ SUR1 serves as a target for sulfonylurea drugs, such as glimepiride and glibenclamide, widely used to promote insulin secretion for blood glucose control in MODY6 patients. However, some patients exhibit insensitivity to sulfonylureas, and satisfactory blood glucose control can be achieved by switching to metformin or thiazolidinediones.^12^ Due to significant variations in the risk of microvascular and macrovascular complications among MODY6 patients, those with severe diabetic complications typically require insulin therapy. Clinical drug selection for MODY6 patients is diverse and should be tailored to individual circumstances.

Furthermore, *NEUROD1* also plays a crucial role in central nervous system development by regulating genes involved in cell survival, differentiation, and energy balance in the cerebral cortex, cerebellum and hippocampus. Homozygous mutations in *NEUROD1* result in neurological disorders including mental deficiency, hippocampal hypoplasia, hearing loss, and epilepsy.^13^ Several cases of permanent neonatal diabetes and neurological abnormalities caused by homozygous *NEUROD1* mutations have been reported in Japan and Europe.^10^ Heterozygous *NEUROD1* mutations can disrupt insulin synthesis, impair endocrine pancreas cell maturation, and lead to hyperglycemia (MODY6). However, homozygous *NEUROD1* mutations not only engender endocrine dysregulation but also precipitate neurological abnormalities.

This paper has documented a previously unidentified heterozygous nonsense *NEUROD1* gene mutation. Clinical characteristics for MODY6 overlap with T1DM, leading to the misdiagnosis of this case. Genetic testing plays a crucial role in accurately diagnosing MODY6, guiding individualized treatment, and delaying the occurrence of complications.

## AUTHOR CONTRIBUTIONS

Yuwen Li contributed to data curation and writing. Meng Hao completed the image visualization. Huige Shao and Yihu Sun supervise the project. Ting Liu and Qian Wen processed review and editing. All authors have diligently reviewed and approved the manuscript's final version.

## CONFLICT OF INTEREST STATEMENT

The authors declare no conflicts of interest.
